# A Case of Isolated Congenital Stapedial Suprastructure Fixation

**DOI:** 10.1155/2022/8620738

**Published:** 2022-03-19

**Authors:** Jeon Mi Lee, Hyun Jin Lee, Sung Huhn Kim

**Affiliations:** ^1^Department of Otorhinolaryngology, Ilsan Paik Hospital, Inje University College of Medicine, Goyang, Republic of Korea; ^2^Department of Otorhinolaryngology-Head and Neck Surgery, Incheon St.Mary's Hospital, College of Medicine, The Catholic University of Korea, Seoul, Republic of Korea; ^3^Department of Otorhinolaryngology, Yonsei University College of Medicine, Seoul, Republic of Korea

## Abstract

We report a case of conductive hearing loss caused by isolated congenital stapedial suprastructure fixation with normal footplate mobility. A 60-year-old woman visited the clinic for right-sided mixed hearing loss. Exploratory tympanotomy revealed a bony synostosis between the stapedial suprastructure and promontory, while all the ossicles were present and normally shaped. As the bony synostosis was separated, the stapes became mobile. This is the first report in the medical literature of this congenital ear anomaly. This case also illustrates that stapedial fixation can occur in the suprastructure as well as in the footplate; thus, one must be mindful of this when performing exploratory tympanotomy for stapes fixation.

## 1. Introduction

Congenital abnormalities of the ears are broadly classified into major and minor anomalies. While the major anomalies involve the external ears, the minor anomalies affect the middle ear structures only. Various minor anomalies have been reported, and several attempts have been made to classify them. The most widely accepted classification was proposed by Cremers and Teunissen based on the surgical findings of 104 minor anomalies, the largest number of cases reported to date [1]. They grouped the minor anomalies into four classes based on surgical findings and reconstructive techniques. All isolated congenital stapes ankyloses were grouped into class 1. However, various types of isolated congenital stapes ankylosis have been reported, and Nandapalen and Tos further proposed subclassification of these based on the developmental pathology, type of corrective surgery required, and functional outcomes [2]. According to the classification, isolated congenital stapes ankylosis is largely divided into two categories, footplate fixation and suprastructure fixation. While footplate fixation is commonly encountered [3], suprastructure fixation is extremely rare, and fewer than 25 cases have been reported since 1957 [4].

Many forms of minor anomalies exist. Here, we present a rare case of isolated stapedial suprastructure fixation with normal footplate mobility caused by bony synostosis between the posterior crura and promontory. This study was approved by the Institutional Review Board of the authors' institute (2021-02-023). To our knowledge, this is the first report of this entity in medical literature.

## 2. Case Presentation

A 60-year-old woman presented to our clinic for nonprogressive hearing loss in the right ear since childhood. There was no history of trauma, otitis, associated tinnitus, or vertigo. There was no history of previous ear surgery, and the family history was noncontributory. Examination revealed a normal-appearing external ear, external auditory canal, and tympanic membrane. A pure-tone audiogram showed a severe mixed hearing loss on the right, whereas the left ear showed a normal hearing level. Temporal bone computed tomography (CT) detected no abnormalities in the middle and inner ear.

**Figure 1:**
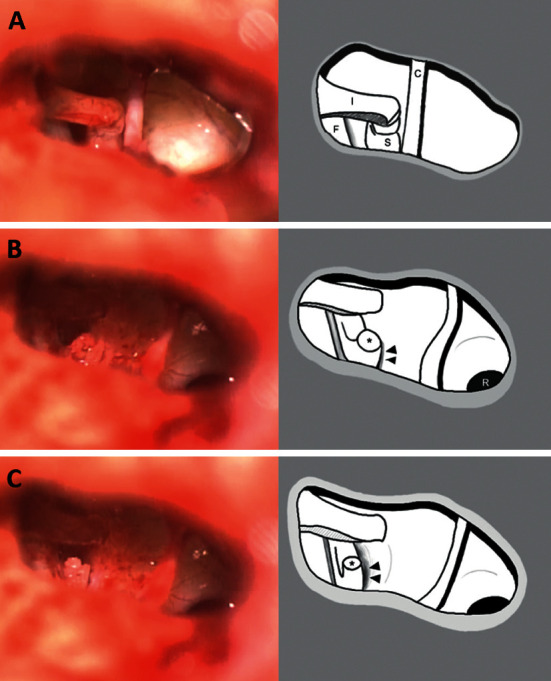
Operative findings. All ossicles were normally shaped (a). Part of the stapedial suprastructure was removed, and the posterior crura showed bony synostosis with the promontory ((b) arrow head). The bony synostosis was separated, and the stapedial footplate became mobile (c). I, incus; F, facial nerve; S, stapes; C, chorda tympani; R, round window; ^*∗*^posterior crura (cut).

A clinical diagnosis of ossicular fixation was suspected, and an exploratory tympanotomy was conducted via the endaural approach under local anesthesia. No ossicles were absent or abnormally shaped (Figure 1(a)). Malleus and incus were typically mobile, but the stapes was fixed. Regarding it as a congenital stapedial footplate fixation, stapedotomy was planned to restore hearing. After cutting the stapedial tendon and separating the incudo-stapedial joint, part of the stapedial suprastructure was removed by cutting the anterior and posterior crura with crurotomy scissors. The stapes remained fixed at that point, and the remnant of the posterior crura showed bony synostosis with the promontory (Figure 1(b)). The bony synostosis was easily separated by applying slight force using a pick, and the separated stapes then became mobile (Figure 1(c)). When tapping the stapes with the pick, the patient could sense the tapping loudly. A total ossicular replacement prosthesis (TORP) was positioned on the stapedial footplate, and harvested tragal cartilage was interpositioned between the TORP and the tympanic membrane. The patient stated that sounds could be heard well, and the operation was completed with gel foam packing of the external canal.

**Figure 2:**
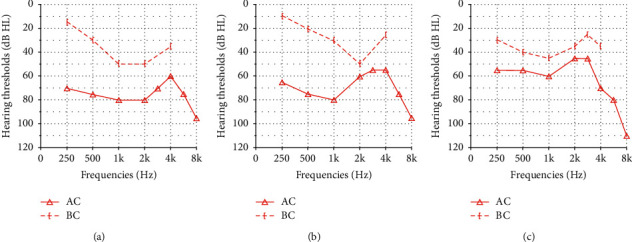
Pure-tone audiometric results preoperation (a), postoperation (b), and after revision surgery (c). AC, air conduction thresholds; BC, bone conduction thresholds.

However, a pure-tone audiogram performed 2 months after surgery showed only slight changes (Figures 2(a) and 2(b)), and the patient underwent revision surgery. On exploration, the malleus and incus were still normal in their shape and mobility, but the remnant stapes and surrounding granulation tissue were severely adhesive. Granulation tissue surrounding the stapedial footplate was carefully dissected to expose the footplate, but it was found to be firmly fixed. Unlike the first surgery, the patient could not sense sound when the footplate was tapped. After checking it several times, regarding it as a stapedial footplate fixation, stapedotomy was performed for hearing restoration. An opening was created at the stapedial footplate using a laser, and the long process of the incus and the stapedial footplate was connected with a piston. A pure-tone audiogram was performed 2 months after surgery, and it showed a decreased air-bone gap and improved air conduction thresholds (Figure 2(c)).

## 3. Discussion

The present case demonstrated isolated congenital stapes ankylosis with suprastructure fixation. While all the middle ear structures were normally shaped, a bony synostosis between the posterior crura and promontory caused fixation. There have been a few presented cases of stapes ankylosis with stapes-promontory fixation, but the fixations were caused mainly by bony bridges between the structures [3, 5]. Only one case of bony synostosis between the stapes and promontory has been reported, but it presented with a deformed stapes [2]. Thus, to our knowledge, ours is the first report of this entity.

**Figure 3:**
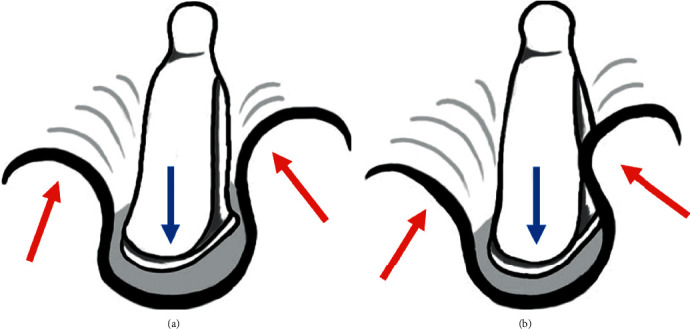
The proposed theory for congenital stapedial suprastructure fixation with normal footplate mobility. During development, the stapes enlarges medially toward the otic capsule, and the otic capsule enlarges laterally toward the stapes (a). The bulging mesenchyma of the otic capsule makes direct contact with the developing stapedial mesenchyma (b).

A possible causal theory has been proposed based on stapedial development [4]. During development, the stapes and otic capsule enlarge in opposite directions; the stapes enlarges medially toward the otic capsule, and the otic capsule enlarges laterally toward the stapes (Figure 3(a)). As they are growing, the bulging mesenchyma of the otic capsule makes direct contact with the developing stapedial mesenchyma (Figure 3(b)). These structures then fuse and later undergo ossification, leading to stapes-promontory fixation.

In cases of stapedial suprastructure fixation with a mobile footplate, division of the bony synostosis would be a treatment of choice for the correction of hearing. Indeed, there have been a few case reports of hearing improvement by simply disconnecting the bony bridge between the stapedial suprastructure and the surrounding structures [2, 3]. In the present case, hearing improvement was expected because a mobile footplate was noted, and the patient subjectively sensed hearing improvement during the procedure. However, the 2-month postoperative pure-tone audiogram revealed no improvement in air conduction thresholds, and a previously absent fixed stapedial footplate was found.

There have been several cases in which a clearly mobile stapes was confirmed during middle ear surgery, and temporary hearing gain was also achieved, but a bony fixation of the stapes necessitating a stapedectomy was found in revision surgery [6]. It was found to be in as many as 19 of 112 cases (17%), and Shea explained this phenomenon as a postinflammatory osteogenic fixation [6]. The trauma from vigorous footplate manipulation during surgery could damage the stapediovestibular joint, and the damage could bring about degenerative arthritis in genetically predisposed individuals. Degenerative changes in the joint and the eventual erosion of the underlying bones result in the growth of new bone to repair the erosions. As this vicious cycle develops, the stapedial footplate becomes fixed. However, the new bone is not abnormal, but identical to otosclerotic bone [7]; thus, hearing gain from conventional stapes surgery can be achieved, as in the present case. In the present case, it was suspected that the fixation of the stapes was newly developed through careful confirmation during two surgeries. Still, the possibility for otosclerosis cannot be completely excluded. Postinflammatory osteogenic stapes fixation probably occurs more frequently than reported since it is difficult to distinguish from concomitant otosclerosis.

This case report not only presents a new congenital middle ear anomaly but also suggests some important points when exploratory tympanotomy or any middle ear surgery for hearing correction are under consideration. In the present case, stapedial footplate fixation was strongly suspected before the first surgery for several reasons: (1) a Carhart notch in the audiogram, (2) normal shapes of middle ear structures on CT scan, (3) absence of previous ear surgery or infection history, and (4) the high incidence of stapedial footplate fixation among isolated middle ear anomalies [8]. However, the Carhart notch is not limited to cases with stapedial footplate fixation; it can be present in cases with any fixation of middle ear structures [9]. Therefore, one must keep in mind that stapes fixation with a mobile footplate can be encountered. In addition, the chances of postinflammatory osteogenic changes after middle ear surgery always exist, and they can be corrected with the classical stapedotomy. However, one should recognize that the degree of hearing improvement might be insufficient compared to that in primary cases [10]. It might be frustrating if hearing deteriorates even after middle ear surgery is performed for hearing improvement. However, with the possibility of postinflammatory osteogenic changes in mind, if stapedial footplate fixation is suspected, reoperation can be carefully but actively considered.

We have presented a rare case of congenital stapedial suprastructure fixation with normal footplate mobility, which has never been reported in the literature. When performing exploratory tympanotomy for stapes fixation, one must keep in mind that fixation can occur in the suprastructure as well as in the stapedial footplate.

## Data Availability

All relevant data are within the article.
